# Preeclampsia Correlates with an Increase in Cannabinoid Receptor 1 Levels Leading to Macromolecular Alterations in Chorionic Villi of Term Placenta

**DOI:** 10.3390/ijms232112931

**Published:** 2022-10-26

**Authors:** Marta Lombó, Christian Giommi, Michela Paolucci, Valentina Notarstefano, Nina Montik, Giovanni Delli Carpini, Andrea Ciavattini, Antonio Ragusa, Francesca Maradonna, Elisabetta Giorgini, Oliana Carnevali

**Affiliations:** 1 Department of Life and Environmental Sciences, Università Politecnica delle Marche, 60131 Ancona, Italy; 2 Department of Molecular Biology, Faculty of Biology and Environmental Sciences, Universidad de León, 24071 León, Spain; 3 Department of Odontostomatological and Specialized Clinical Sciences, Università Politecnica delle Marche, 60020 Ancona, Italy; 4 Department of Obstetrics and Gynecology, Università Campus Bio Medico di Roma, 00128 Roma, Italy; 5 INBB—Consorzio Interuniversitario di Biosistemi e Biostrutture, 00136 Roma, Italy

**Keywords:** preeclampsia, endocannabinoid system, CB1, collagen, lipid peroxidation, FTIRI

## Abstract

Preeclampsia is a human pregnancy-specific disease characterized by abnormal placentation that usually presents with maternal hypertension and proteinuria. The main hallmark of preeclampsia, impaired trophoblast migration, and the subsequent disruption of uterine arteries remodeling lead to several molecular alterations in the placental compartments with those occurring in the chorionic villi being of the utmost importance. Given the essential role of the endocannabinoid system during preimplantation and trophoblast migration, we have combined the histological and hyperspectral imaging analyses to shed light on the involvement of two cannabinoid receptors in the macromolecular alterations related to preeclampsia. The results obtained by immunohistochemistry showed a significant increase in the protein levels of cannabinoid receptor 1 (CB1) in the preeclamptic chorionic villi. However, no changes were reported regarding transient receptor potential vanilloid 1 (TRPV-1) levels either in the bulk placental samples or chorionic villi when comparing control and preeclamptic patients. Histological analysis and Fourier-transform infrared spectroscopy (FTIRI) showed an increase in collagen deposition together with higher levels of lipid peroxidation and phosphorylated compounds in the pathological villi. Since CB1 enhancement has been described as promoting fibrosis and oxidative stress in several tissues, we proposed that the higher receptor abundance in preeclampsia could be triggering similar molecular effects in preeclamptic term placentas.

## 1. Introduction

Preeclampsia is a severe trophoblast-related disorder affecting up to 8% of all pregnant women and leading to over 50,000 maternal and 500,000 fetal deaths worldwide every year [1,2]. It is characterized by the occurrence of hypertension (blood pressure > 140 mm Hg and/or diastolic blood pressure > 90 mm Hg) and significant end-organ dysfunction with or without proteinuria (>300 mg/day) after 20 weeks of gestation or postpartum in a previously normotensive patient [3]. Depending on its clinical manifestations, it can be classified into: early-onset or placental preeclampsia (occurring before 34 weeks), which is more often related to intrauterine growth restriction, and late-onset or maternal preeclampsia (occurring after 34 weeks) [4].

The first stage of preeclampsia pathogenesis results from the failure of proliferative epithelial trophoblasts to become invasive endothelial cells, impairing proper spiral artery remodeling. Consequently, the narrow maternal vessels formed induce relative placental ischemia and, eventually, raise the levels of oxidative stress. In the second stage, the release of several molecules and factors from the incompletely implanted placenta triggers endothelial cell activation and clinical manifestations [5]. Preeclamptic placentas are characterized by vascular lesions, chronic inflammation, peri-villous coagulation, and villous fibrosis. Chorionic villi (CV) include the trophoblasts and villous stroma, a structure derived from the extraembryonic mesoderm that contains reticulum and mesenchymal cells as well as fibroblasts [6]. Even though preeclampsia is predominantly described as a trophoblast dysfunction, the fibrosis of villous stroma is also a defining feature of this disease [7]. Bearing in mind that some women had been diagnosed with preeclampsia in the absence of hypertension or proteinuria, the standard clinical definition needs a revision [8]. It is noteworthy that changes in the endocannabinoid system (ECS) have been also associated with preeclampsia [9]. The ECS is a signaling system that comprises endocannabinoids (endogenous bioactive lipids, ECs), cannabinoid receptors (CBs), and the enzymes involved in their metabolism. Besides the canonical CB1 and CB2, other EC receptors have been described, such as the transient receptor potential vanilloid 1 (TRPV1) [10], peroxisome proliferator-activated receptors (PPAR) [11,12,13] as well as G-protein-coupled receptors GPR55 [14] and GPR119 [15]. In human term gestation, an increase in plasma levels of the most studied endocannabinoid, N-arachidonoylethanolamine (anandamide, AEA) has been reported [16]. Compared to other reproductive tissues, the placenta produces the highest levels of AEA that, after its synthesis, predominantly binds to CB1, its most abundant receptor in the human term placenta [17]. The desensitization of CB1, occurring by endocytosis, is a crucial process since an aberrant activation of this receptor has been linked to different pathologies [18,19,20]. As far as CB1 levels in preeclampsia are concerned, the results are not consensual: some authors have confirmed an increase of CB1 in pathological placenta [21], whereas others have reported no differences between control and preeclamptic samples [22]. Regardless of these inconclusive findings, an epidemiological study carried out in central Europe showed that a single-nucleotide polymorphism in the gene encoding CB1 (*CNR1*) was correlated with preeclampsia [23]. As for TRPV-1, there is only one study in which both transcript and protein levels in preeclamptic placenta are analyzed [24]. Therefore, the role of endocannabinoid receptors in the onset and progression of preeclampsia is still far from being unveiled.

Preeclampsia has been described as a human-specific disease, likely resulting from the high ratio of brain:body weight of the human fetus, which requires 60% of the nutritional exchange from the mother in the third trimester [25]. Albeit indispensable to understanding some pathogenic pathways of preeclampsia, the use of other mammal models may not be so adequate for studying a multifactorial human-specific disease [26]. This fact, together with the ethical challenges concerning the examination of pregnant women, has hindered the comprehension of both its etiology and treatment. Unraveling the molecular mechanisms behind preeclampsia may hold the key to accurately managing the pregnancy and labor complications derived from this disease. In this scenario, we hypothesize that changes in the endocannabinoid receptors CB1 and TRVP-1 could be related to the macromolecular alterations described in preeclamptic conditions.

## 2. Results

### 2.1. Preeclampsia Increases CB1 Levels in the Chorionic Villi

**Figure 1:**
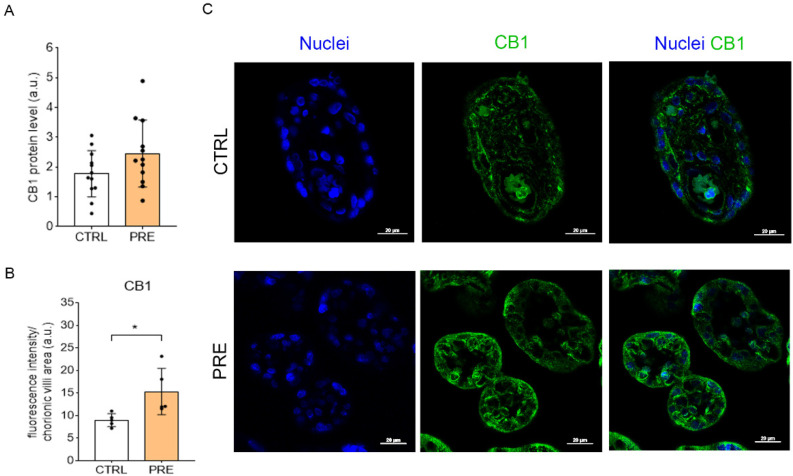
Relative levels of CB1 in the two experimental groups. (**A**): Analysis of the CB1 levels in placenta slices by western blot. Bars represent the densitometric analysis of CB1 (53 kDa)/β-actin (42 kDa) ratio ± SD of twelve independent replicates (*n* = 12). (**B**): Analysis of the CB1 levels in chorionic villi by immunohistochemistry. Bars represent the fluorescence intensity per area of chorionic villi ± SD of five independent samples (*n* = 5). In confocal images (**C**), nuclei are marked with DAPI (blue) and CB1 is marked in green (Alexa Fluor^®^ 488); scale bar = 20 µm. Asterisk indicates significant differences (* = *p* < 0.05) when comparing preeclamptic samples to the control group.

CB1 levels in human term placenta were measured in control (CTRL) and preeclamptic (PRE) patients. Although the results did not show any statistical differences between the two groups (*p* = 0.0995; *n* = 12), protein levels of CB1 were slightly increased in PRE placenta (Figure 1A). Considering not only the great differences in both cell type composition and transcriptional activity found throughout the placental compartments [27], but also the high expression of CB1 in the CV [28], the levels of the receptor were specifically measured in these structures. The immunohistochemistry analysis showed a significant increase of CB1 levels in the CV of preeclamptic placenta (Figure 1B) (*p* = 0.0287; *n* = 5), as well as a predominant expression of CB1 in the cytoplasm or in the apical membrane of CTRL and PRE syncytiotrophoblast (SCTB), respectively (Figure 1C).

### 2.2. Preeclampsia Does Not Change TRPV-1 Levels in Placenta

**Figure 2:**
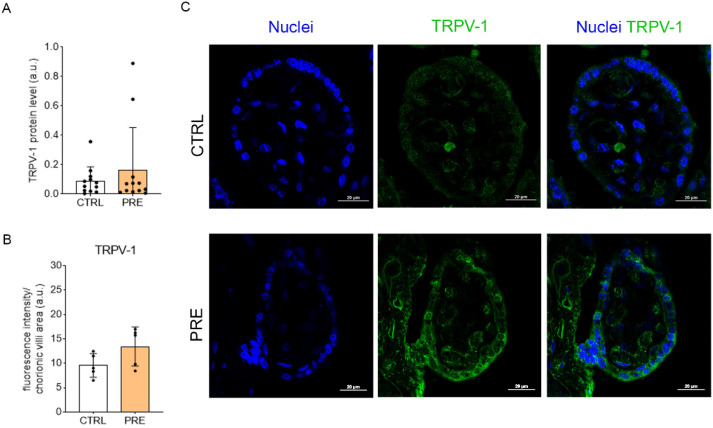
Relative levels of TRPV-1 in the two experimental groups. (**A**): Analysis of the TRPV-1 levels in placenta slices by western blot. Bars represent the densitometric analysis of TRPV-1 (100 kDa)/β-actin (42 kDa) ratio ± SD of twelve independent replicates (*n* = 12). (**B**): Analysis of the CB1 levels in chorionic villi by immunohistochemistry. Bars represent the fluorescence intensity per area of chorionic villi ± SD of five independent samples (*n* = 5). In confocal images (**C**), nuclei are marked with DAPI (blue) and TRPV-1 is marked in green (Alexa Fluor^®^ 488); scale bar = 20 µm.

As for the levels of TRPV-1, preeclampsia did not alter either the levels of the receptor in the whole placenta (*p* = 0.3906; *n* = 12) (Figure 2A) nor the specific levels in the CV (*p* = 0.0999; *n* = 5) (Figure 2B). Regarding its localization, TRPV-1 was mostly found in the cytoplasm of SCTB in CTRL placenta, whereas it was also expressed in the apical membrane of SCBT in PRE placenta (Figure 2C).

### 2.3. Infrared Spectra Analysis and Multivariate Analysis

**Figure 3:**
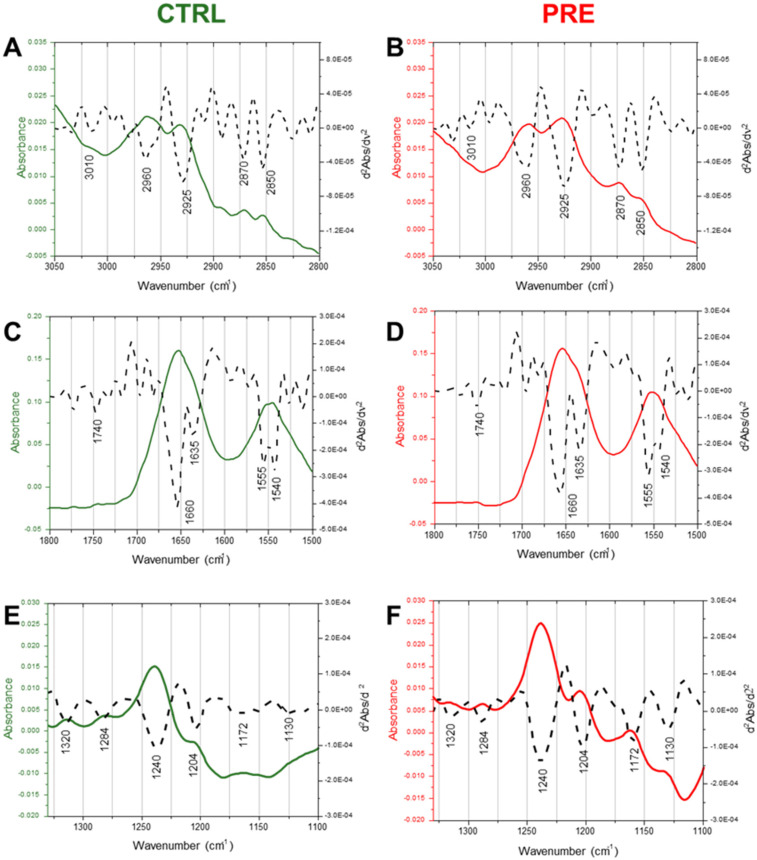
Infrared spectra. Average spectra of control (CTRL, green lines) and pre-eclampsia (PRE, red lines) samples, in the 3050–2800 cm^−1^ (**A**,**B**), 1800–1500 cm^−1^ (**C**,**D**), and 1330–1100 cm^−1^ (**E**,**F**) spectral ranges. Spectra are reported in absorbance (colored continuous lines) and second derivative modes (black dotted lines).

The average IR spectra of CTRL and PRE experimental groups are reported in Figure 3, both in absorbance (continuous colored lines) and in second derivative (dotted black lines) modes in the 3050–2800 cm^−1^, 1800–1500 cm^−1^, and 1330–1100 cm^−1^ spectral ranges. Labels along the second derivative spectra indicate the most relevant IR bands: 2960 cm^−1^ and 2870 cm^−1^ (asymmetric and symmetric stretching vibrations of CH_3_ groups); 2925 cm^−1^ and 2850 cm^−1^ (asymmetric and symmetric stretching vibrations of CH_2_ groups); 1740 cm^−1^ (stretching vibration of C=O ester moieties in triglycerides and phospholipids); 1660 cm^−1^ and 1635 cm^−1^ (Amide I band components of proteins, AI) [29,30,31]; 1555 cm^−1^ and 1540 cm^−1^ (Amide II band components of proteins, AII) [29,30,31,32]; 1320 cm^−1^ (collagen’s α-helix secondary structures) [33,34,35]; 1284 cm^−1^ and 1240 cm^−1^ (collagen’s triple helix) [33,34,35]; 1204 cm^−1^ (collagen’s amino acids lateral chains) [33,34,35,36]; 1172 cm^−1^ phosphodiester bond (COP) [34] and 1130 cm^−1^ [32].

**Figure 4:**
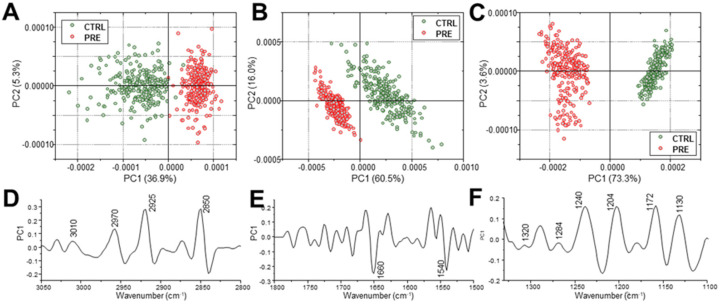
Multivariate analysis on IR spectra. PCA scores plots and corresponding PC1 loadings calculated for CTRL and PRE experimental groups in the following regions of interest (ROI): 3050–2800 cm^−1^ (**A**,**D**), 1800–1500 cm^−1^ (**B**,**E**), and 1330–1100 cm^−1^ (**C**,**F**). For clarity purposes, PCA loadings are plot with different Y scales for the three selected ROI.

To precisely assess the spectral modifications induced by pre-eclampsia on CV, principal component analysis (PCA) was performed, on the three specific ROI: 3050–2800 cm^−1^, 1800–1500 cm^−1^, and 1330–1100 cm^−1^ (Figure 4). A clear segregation was found between CTRL and PRE groups in all the performed comparisons. The analysis of Principal Component 1 (PC1) loadings highlight the most discriminant spectral features labelled in Figure 4D–F.

### 2.4. Collagen Deposition in the Chorionic Villi Is Increased in Preeclamptic Placenta

**Figure 5:**
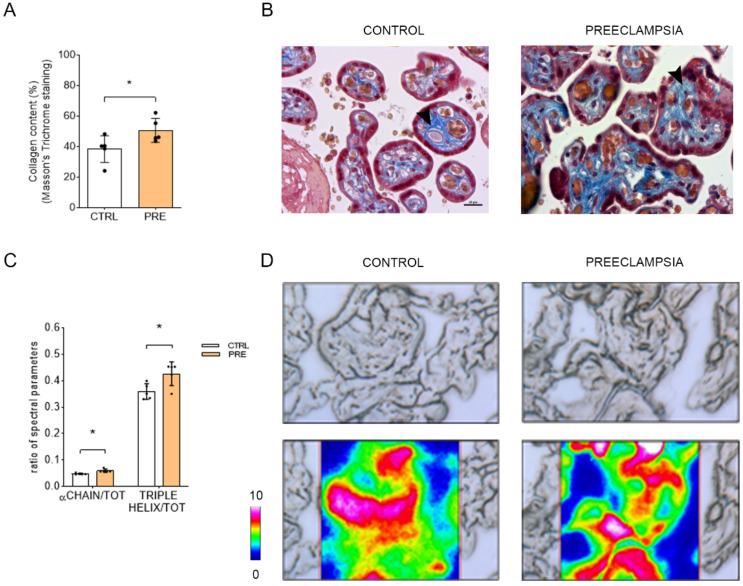
Histological and FTIRI analysis of collagen in the two experimental groups. Analysis of collagen deposition in CV sections using Masson’s trichrome staining (**A**,**B**). Bars represent collagen content in the two conditions expressed as mean ± SD of five independent samples (*n* = 5). Histological images show the collagen stained in blue (arrowhead) inside the CV stroma. Analysis of collagen deposition in CV section by means of FTIRI (**C**,**D**). Bars represent the ratio of the following spectral parameters: α chain/TOT, relative amount of α chain secondary structures in collagen; Triple helix/TOT, relative amount of triple helix structures in collagen. Results are reported as mean ± SD of five independent samples (*n* = 5), and asterisks indicate significant differences between CTRL and PRE groups (* = *p* < 0.05). In false color images, the topographical distribution of protein within the mapped area in the Amide III was reported with absorbances ranging from pink representing the highest absorbance and blue representing the lowest absorbance.

The collagen deposition in the CV was evaluated using a Masson’s trichrome staining (Figure 5A,B) and Fourier transform infrared imaging (FTIRI) analysis (Figure 5C,D). The histochemistry assessment revealed an increase in the deposition of collagen in PRE placentas compared to CTRL ones (*p* = 0.00355; *n* = 5). In addition, FTIRI demonstrated a significant increase of α chain/Total Protein (TOT) (*p* = 0.0153; *n* = 5) and triple helix/TOT (*p* = 0.0270; *n* = 5) in the Amide III region of PRE placentas compared to CTRL ones, suggesting alterations in the organization of collagen in the CV stroma.

### 2.5. Preeclampsia Does Not Change Lipid Composition but Triggers an Increase of Lipid Peroxidation

**Figure 6:**
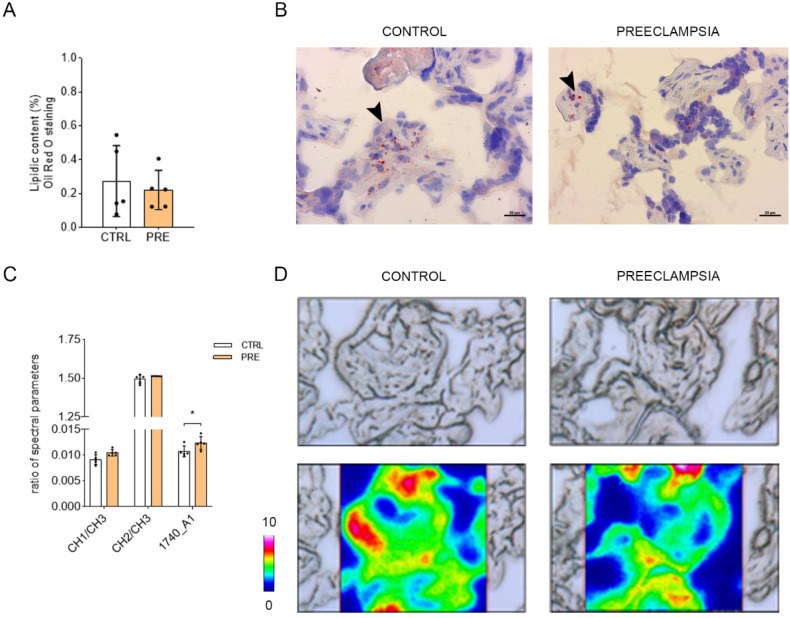
Histological and FTIRI analysis of lipids in the two experimental groups. Analysis of lipid content in CV section using oil red O staining (**A**,**B**). Bars represent lipid content in the two conditions expressed as mean ± SD of five independent samples (*n* = 5). Histological microphotographs show the lipid droplets stained in red (arrowhead) inside the CV. Analysis of lipid content and peroxidation in CV section by means of FTIRI (**C**,**D**). Bars represent the ratio between the following spectral parameters: CH2/CH3, length of lipid alkyl chains; CH/CH3, unsaturation degree in lipid alkyl chains; 1740/AI, ester groups in lipid alkyl chains. Results are reported as mean ± SD of five independent samples (*n* = 5), and asterisks indicate significant differences between CTRL and PRE groups (* = *p* < 0.05). False-color images showed the topographical distribution of lipids within the mapped area with absorbance ranging from pink representing the highest absorbance and blue representing the lowest absorbance.

Placental lipid content was evaluated within the CV at histological level by oil red O staining (*p* = 0.6353; *n* = 5) (Figure 6A,B) and FTIRI analysis (Figure 6C,D). None of the assessments showed any changes in the lipid content or composition (band area ratio of CH/CH3 representing the saturation of alkyl chains (*p* = 0.0733; *n* = 5), and CH2/CH3 indicating the length and saturation of alkyl chains (*p* = 0.3989; *n* = 5). Despite this, the analysis of IR spectra revealed a significant increase in lipid peroxidation, as indicated by the 1740/AI band area ratio (*p* = 0.0309; *n* = 5).

### 2.6. Preeclamptic Chorionic Villi Characterized by an Increase in Phosphoester Bonds 

**Figure 7:**
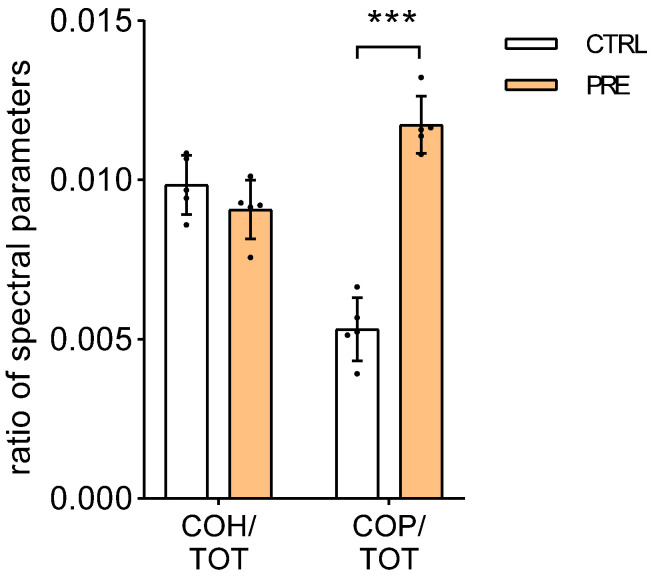
Analysis of relative amount of glycosylated (COH) and phosphorylated (COP) compounds in CV by means of FTIRI. Bars represent the ratio between the following spectral parameters: COH/TOT an COP/TOT. Results are reported as mean ± SD of five independent samples (*n* = 5) and asterisks indicate significant differences between CTRL and PRE groups (*** = *p* < 0.001).

Eventually, the relative levels of glycosylated and phosphorylated compounds were assessed in the CV. Even though the levels of glycosylated compounds did not vary between CTRL and PRE (*p* = 0.232; *n* = 5), the phosphorylated compounds were significantly increased in the CV of PRE placenta (*p* = 0.001; *n* = 5) (Figure 7).

## 3. Discussion

Pregnancy relies on the homeostasis and communication of a wide range of cells belonging to different placental compartments. Therefore, to shed light on the causes underlying the disruption of maternal–fetal dialogue that results in adverse pregnancy outcomes, such as preeclampsia, more accurate assessments than the bulk analyses need to be performed. In that regard, the FTIRI analysis of tissue samples allows a 2D morpho-chemical correlation between the histological and spectroscopic data by topographically detecting changes in the biochemical composition and/or conformation of the biomolecules of interest, on the same tissue section and without any label. Moreover, FTIRI is a valuable and reliable tool for the study of the alterations in the secondary structure of proteins (including collagen) as biomarkers of some pathological conditions, by the thorough spectral analysis of Amide bands [34,36]. In this study, immunohistochemistry was carried out to analyze the specific levels of the endocannabinoid receptors in the CV, and by combining this technique with histomorphometry and a high-throughput technique, the FTIRI, we have investigated their potential link to the macromolecular alterations occurring in the CV of preeclamptic placenta. Bearing in mind that the use of placenta for research purposes can pose some problems for experimental reliability and reproducibility, we have tried to minimize this limitation by collecting the samples in a specific region: nearby the umbilical cord of the fetal side. For this reason, we have also analyzed approximately half of placentas from natural delivery and half from Cesarean section, since the former may affect placenta structure, whereas the latter can trigger changes at metabolic, gene expression, and protein level [37]. 

During normal pregnancy, placental cytotrophoblasts invade the maternal uterine wall and spiral arteries, but this process is interrupted in preeclamptic conditions [38]. In mice, it has been demonstrated that the tightly regulated migration of trophoblasts can be impaired by both overexpression and repression of CB1, leading to poor placentation [39]. Considering that preeclampsia is a severe trophoblast-related disorder, we analyzed the levels of CB1 in the placenta of control and preeclamptic patients. Our findings showed no changes in the total receptor levels (analyzed by western blot) when comparing control and preeclamptic groups, most likely due to the differential expression of CB1 throughout the placental compartments [17], which could bias this outcome. In that regard, the previous results obtained by western blot were controversial: some authors have not reported any alterations in the overall levels of CB1 in preeclamptic placentas [22], whereas others have demonstrated higher levels of the receptor in the pathological samples [21]. CB1 is highly expressed in the SCTB layer as well as the endothelial cells of blood vessels [22,28], so we performed an immunohistochemical assay to have a deeper insight into its specific expression. In this case, the analysis did show that, in preeclampsia, CB1 levels within the chorionic villi are significantly increased, especially in the apical membrane of SCTB. These results are in accordance with those found by Fugedi and colleagues [21], reporting a qualitatively higher CB1 immunoreactivity in both SCTBs and the mesenchymal core of preeclamptic samples compared to control ones. Another important endocannabinoid receptor is TRPV1, an ion channel also expressed in the placenta where it is involved in the transepithelial transfer of calcium to the fetus, a process requiring a passive/active transport from maternal blood to the cytoplasmic compartment of the SCTB [40,41]. Since the disruption of calcium homeostasis is a hallmark of preeclampsia [42], we investigated the levels of TRPV1 inside the CV of both control and preeclamptic placentas. The previous results regarding TRPV-1 in preeclampsia showed that the increase in mRNA levels of the preeclamptic placentas did not correspond to higher TRPV-1 protein levels [24]. In this study, we have not observed any statistical differences in the TRPV-1 protein levels either in the bulk samples or in the chorionic villi between control and preeclamptic patients. Still, it seems that the location of this receptor follows the same pattern described for CB1, being mainly expressed in the apical membrane of preeclamptic SCTB where it is likely to allow the entrance of calcium from maternal blood.

As mentioned above, impaired trophoblast proliferation and migration can lead to improper remodeling of maternal spiral arteries, which is one of the main factors contributing to preeclampsia. It is noteworthy that an excess of collagen I led to preeclampsia-like features in pregnant mice since it suppresses trophoblast proliferation and invasion through inhibition of ERK phosphorylation and the WNT/β-catenin signaling pathway [43]. These authors also reported an increase in the collagen I deposition as well as a higher gene and protein expression of collagen in the preeclamptic placenta [43]. Collagen deposition has also been evaluated in first-term villi and full-term placentas, and the results obtained by Masson’s trichrome staining and western blot also showed higher levels of collagen in preeclampsia patients [44]. Here, we have studied the collagen I deposition in the chorionic villi of term placenta with a novel approach that combines the histological, histomorphometric, and hyperspectral analyses. Our findings demonstrate not only a significant increase in collagen I deposition in the preeclamptic villi, but also remarkable alterations in the collagen secondary structures: the assessment of the Amide III band (mainly attributed to collagen [33,34,36]) showing higher levels of collagen α chain and collagen triple helix in the pathological villi. Indeed, villous stroma has been described as the main placental compartment affected by the preeclampsia, being fibrosis of the villous stroma one of the most important features of this disease [7]. In mice, genetic and pharmacological inactivation of CB1 has been reported to decrease liver fibrogenesis by reducing the TGF-β1 levels, thus decreasing the accumulation of fibrogenic cells after apoptosis and inhibiting the growth of hepatic myofibroblasts [45]. Likewise, the treatment of skin-derived human fibroblasts with AM251 (a CB1 selective antagonist) has been proven to inhibit both fibroblast differentiation into myofibroblast and collagen deposition [46]. Therefore, the significant increase in collagen deposition we reported in the term chorionic villi of preeclamptic patients might well be a result of the CB1-enhanced expression characteristic of these pathological villi.

Preeclampsia has been also associated with oxidative stress caused by the intermittent arterial blood flow resulting from the impaired remodeling of the maternal spiral arteries [47]. The increased generation of reactive oxygen species (ROS) leads to lipid peroxidation; thus, an increase in placental production of lipid peroxides and thromboxane has been reported in preeclampsia patients [48]. In this work, the analyses performed using oil red O and FTIRI have shown that, despite the lack of changes in lipid composition, preeclamptic CV displayed higher levels of lipid peroxidation. This data supports the fact that, in preeclampsia, the production of lipid peroxides and thromboxane originates from both the trophoblasts cells and villous core compartments [48]. Since CB1 activation has been stated to enhance the ROS production in macrophages, neurons, and cardiomyocytes [18,19,20], there could be a link between the higher levels of CB1 in the villi of preeclamptic patients and the rise in lipid peroxidation as well. 

Along with the increase in collagen deposition and lipid peroxidation, the FTIRI analyses showed much higher levels of phosphorylated compounds, containing a phosphoester bond, in the preeclamptic CV than in the control ones. Among these phosphorylated compounds, phosphocholine levels have been reported to change in a rat model of preeclampsia [49] and in human preeclamptic placenta [50]. The binding of phosphocholine to C-reactive protein localized the SCTB villi cells has been reported to induce arterial hypertension and placental damage [51,52]. Moreover, phosphoesters derived from phosphocholine have been found in 63% of preeclamptic blood plasma [53]. Therefore, our findings support the involvement of phosphoesters in the pathogenesis of preeclampsia. 

Overall, this study demonstrates that the FTIRI analysis is a powerful tool for the identification of some crucial molecules involved in preeclampsia; thus, its application at earlier stages of the disease might improve the diagnosis. Moreover, it highlights the importance of performing specific analyses in the different placental compartments to have a better understanding of the dysregulated molecular pathways involved in pathological conditions, such as preeclampsia. Our findings point to the pivotal role of the endocannabinoid system, in particular CB1 levels, in some of the main molecular alterations related to preeclampsia, such as fibrosis and oxidative stress, providing new insights into the pathogenesis of this disease. Further studies will be needed to assess whether the restoration of CB1 physiological levels could be an effective treatment to mitigate the molecular alterations described in term preeclamptic placenta.

## 4. Materials and Methods

### 4.1. Ethics Declarations and Sample Collection

Term placenta samples were collected shortly after (<30 min) delivery at G. Salesi Hospital, Ancona (Italy). Informed consent was obtained from all subjects involved in the study. The study was approved by local ethical committee (Comitato Etico Regionale Marche, n° CERM 241/2020).

After delivery, small pieces were cut from the fetal side of the placenta, nearby the umbilical cord, under aseptic conditions. Then, one portion of placenta was frozen at −80 °C for western blot and FTIRI analyses, while another portion was fixed in formalin solution (Bio Optica, Milan, Italy) to be subsequently processed for histology and immunohistochemistry.

Diagnosis of preeclampsia was performed according to data present in Table 1. 

### 4.2. Evaluation of Protein Levels by Western Blot

Slices of frozen placenta samples from 12 CTRL and 12 PRE patients were lysed in Hanna’s buffer containing 0.125 M Tris-HCl pH 7.5, 4% (*w/v*) SDS, 20% (*v/v*) glycerol, and 10% (*v/v*) β-mercaptoethanol, supplemented with 1:10 Protease Inhibitor Cocktail (Sigma-Aldrich^®^, Milan, Italy). The lysate was incubated at 100 °C for 5 min and centrifuged at 12,000× *g* for 15 min. The supernatant was collected, and the protein quantity was quantified using Bradford Reagent (Sigma-Aldrich^®^, Milan, Italy) following the manufacturer’s instructions. Proteins were run in SDS-PAGE gel (10% of acrylamide for the analysis of endocannabinoid receptors and 15% for the analysis of antioxidant proteins, loading 25 µg of total protein per well). The proteins were transferred into a nitrocellulose membrane for immunoblotting incubating during 2 h at 250 mA in transfer solution (25 mM Tris, 192 mM glycine, and 20% (*v/v*) ethanol). Blocking was conducted with 5% BSA in TBS-T (0.1% Tween), and membranes were incubated overnight at 4 °C with the corresponding antibodies diluted 1:1000: anti-β-actin (#4967, Cell Signalling TECHNOLOGY^®^, Danvers, USA), anti-CB1 (ab259323, Abcam, Cambridge, UK), and anti-TRPV1 (ab3487, Abcam, Cambridge UK). Incubation with the secondary antibody goat anti-rabbit IgG-HRP (Sigma-Aldrich^®^, Milan, Italy) diluted 1:2500 was performed for 1 h at 30 °C. The chemiluminescent signal was digitalized using Image Lab Software (Bio-Rad, Hercules, Wilmington, DE, USA), whereas the measure of protein levels was carried out using Fiji ImageJ Software (Bethesda, Rockville, MD, USA). The representative images of the western blot bands are shown in Figure S1.

### 4.3. Immunohistochemistry and Image Analysis

Routine histology was carried out using 5 CTRL and 5 PRE placenta. Briefly, placentas were fixed in formalin solution (Bio Optica, Milan, Italy) and paraffin-embedded after dehydration in an ascendant alcohol series (70%, 80%, 95% and 100%; 1 h per step) and immersion in Xylene (Bio Optica, Milan, Italy) for 45 min. Sections of 4 μm in thickness were produced using a rotating microtome (Leica, RM2125RTS, GmbH, Wetzlar, Germany) and, after drying, were de-waxed and rehydrated. For the detection of CB1 and TRPV-1, an antigen retrieval was performed by incubating the slices in a buffer containing 10 mM sodium citrate and 0.05% (*v/v*) Tween at 100 °C for 20 min. After washing with distilled water, the sections were permeabilized in 1% PBS-T (triton X-100) for 20 min. Blocking was carried out in PBS supplemented with 3% BSA and 0.1% (*v/v*) Tween during 1 h in a humid box. Next, sections were incubated overnight at 4 °C with the same antibodies used for the western blot but, in this case, dilute 1:100 in blocking solution. Following three washing steps with PBS, sections were incubated with a Goat Anti-Rabbit IgG H&L (Alexa Fluor^®^ 488; ab150077, Abcam, Cambridge, UK) at 37 °C for 1 h. Slides were washed three times with PBS, and mounted with DAPI-Aqueous Fluoroshield (ab104139, Abcam, Cambridge, UK). The images on slides were taken using a confocal microscope Nikon A1R, whereas the levels of CB1 and TRPV-1 in the chorionic villi were analyzed in 5 different sections per sample using Fiji ImageJ Software. 

### 4.4. Histochemical Analysis of Collagen and Lipid Content

Regarding collagen content analysis, samples were fixed and paraffin-embedded using the same protocol described for immunohistochemistry. Then, four histological sections of 4 μm in thickness were cut from 5 CTRL and 5 PRE placenta samples at a distance of 100 μm among sections to obtain representative portions of the samples and then stained with Masson’s trichrome (Bio Optica, Milan, Italy) following the manufacturer’s instructions. 

As for lipid staining, a portion of the −80 °C-stored samples were included in Killik O.C.T. (Bio Optica, Milan, Italy) and cut at a thickness of 8 μm in a cryostat (MC4000, Histo-Line) at −26 °C, producing four sections at a distance of 100 μm that were disposed on a gelatin-coated slide. Frozen sections were post-fixed using paraformaldehyde (PFA) 4% for 15 min in a humid environment, washed in deionized water and left to dry for an hour. Sections were then rinsed in 60% (*v*/*v*) isopropanol for 5 min and then stained for 15 min with oil red O solution, which was prepared by dissolving 0.5 g of oil red O powder in 100 mL of 100% isopropanol, forming a stock solution, and then diluted with deionized water obtaining a working solution of oil red O in 60% isopropanol that was filtered (0.4 μm) before being used. Sections were then rinsed in 60% isopropanol and counterstained with Mayer’s Hematoxylin for 5 min and mounted with 90% glycerol.

All samples processed for histology were visualized under an optical microscope, the Zeiss Axio Imager A.2 (Zeiss, Oberkochen, Germany), and 5 images were taken using an Axiocam 503 camera for every sample and then analyzed using Fiji ImageJ Software.

### 4.5. FTIRI Analysis 

Portions of placentas from 5 CTRL and 5 PRE groups were collected and immediately cryopreserved at −80 °C. Then, from each sample, three sections (~10 µm thick) were cut using a cryomicrotome; sections were deposited onto CaF_2_ optical windows (1 mm thick, 13 mm diameter) and left to air-dry for 30 min.

FTIRI measurements were performed at the ARI Lab of the Department of Life and Environmental Sciences, Polytechnic University of Marche. A Bruker INVENIO interferometer, coupled with a Hyperion 3000 Vis-IR microscope and equipped with a Focal Plane Array (FPA) detector operating at liquid nitrogen temperature (Bruker Optics, Ettlingen, Germany) was used for the acquisition of IR images. First, to identify the areas of interest, a microphotograph of each section was acquired by using the television camera. Then, on each section, IR images were acquired in transmission mode with a 15× condenser/objective in the 4000–800 cm^−1^ spectral range, with a spectral resolution of 4 cm^−1^. Each IR image was 164 × 164 µm size and was composed by 4096 pixel/spectra; each pixel/spectrum was the result of 256 scans and the spatial was 2.56 × 2.56 µm. Before starting each sample acquisition, the background spectrum was acquired, with the same parameters, on a clean portion of the CaF_2_ optical window. All IR images were pre-processed using Atmospheric Compensation (to avoid carbon dioxide and water vapor atmospheric contributions), and Vector Normalization (to correct differences in section thickness) routines (OPUS 8.1 software package, Bruker Optics, Ettlingen, Germany).

To pinpoint the topographical distribution of specific macromolecular components, false-color images were generated by integrating pre-processed IR images under 3000–2800 cm^−1^ (stretching vibrations of CH_2_ and CH_3_ groups in lipid alkyl chains; LIPIDS); 1800–1500 cm^−1^ (Amide I and II bands of proteins, vibrational modes of peptide linkage; PROTEINS); and 1350–1100 cm^−1^ (vibrational modes of Amide III band, mainly attributed to collagen; COLLAGEN). A false-color scale was employed: white/light pink indicated zones with the highest absorbance values, while black/dark blue the zones with the lowest ones.

Based on microphotographs, mapping subsets of spectra representative of chorionic villi were extracted from each section. The extracted spectra did not display scattering background and spectral artifacts, and were, hence, only submitted to two-points baseline linear fitting and vector normalization (OPUS 8.1 software, Bruker Optics, Ettlingen, Germany) [31]. Then, the processed spectra were converted in second derivative mode (Savitzky–Golay filter, 9 points of smoothing) and subjected to multivariate analysis, with no further preprocessing. 

The extracted spectra of CTRL and PRE groups were also averaged and submitted to peak fitting procedure in the following regions of interest (ROI): 3050–2800 cm^−1^ (stretching vibrations of CH_2_ and CH_3_ groups in lipid alkyl chains); 1800–1500 cm^−1^ (Amide I and II bands of proteins, vibrational modes of peptide linkage); 1350–1100 cm^−1^ (vibrational modes of Amide III band, mainly attributed to collagen, and phosphate and carbohydrates groups). For each ROI, the number and position of the underlying bands were identified by second derivative minima analysis and fixed during fitting procedure with Gaussian functions (GRAMS/AI 9.1, Galactic Industries, Inc., Salem, NH, USA). The integrated areas of definite underlying bands were used to calculate specific band area ratios.

### 4.6. Statistical Analysis

Statistical analyses were performed using Graph Pad Prism V8.0.1. (GraphPad Software, Inc., San Diego, CA, USA). The normality of the data was checked using the Shapiro–Wilk test and the appropriate statistical assay was accordingly applied. For parametric data an unpaired *t* test with Welch’s correction was used, whereas, for non-parametric data, a Mann–Whitney test was applied. Statistical significance was set at *p*  <  0.05 for all the tests.

Regarding the IR data, the differences between groups were statistically analyzed with the software package Graph Pad Prism V8.0.1. (GraphPad Software, San Diego, CA, USA). All data were presented as mean ± SD. Statistical significance among groups was evaluated using Student’s *t*-test. Statistical significance was set at *p* < 0.05. Moreover, a PCA was performed as an unsupervised multivariate approach to compare the spectral profiles of CTRL and PRE experimental groups (OriginPro 2018b software, OriginLab Corporation, Northampton, MA, USA) [29,31].

## Figures and Tables

**Table 1:** Clinical features of patients and diagnostic parameters. Data are reported as n (%), mean ± SD of twelve independent samples (*n* = 12). *p* < 0.05 was considered as statistically significant. * corresponds to *p* < 0.05, and **** corresponds to *p* < 0.0001.

	CTRL	PRE
**Number of pregnant women**	12	12
**Mode of delivery (%)**		
Natural birth	6 (50%)	7 (58.3%)
Cesarean section	6 (50%)	5 (41.7%)
**Parity**		
Multiparous	9 (75%)	7 (58.3%)
Primiparous	3 (25%)	5 (41.7%)
**Maternal age (years)**	34.2 ± 3.1	37.5 ± 4.4
**Gestational age (weeks)**	39.4 ± 1.5	38.3 ± 1.4
**Mean blood pressure (mmHg)**		
Systolic	116.7 ± 10.9	156.1 ± 21.06 ****
Diastolic	70.9 ± 8.6	99.08 ± 8.7 ****
**Signs of organ damage (%)**		
Proteinuria 24 h ≥ 0.3 g/dL and/or Protein/creatinine ratio ≥ 30 mg/mmol and/or dipstick 2+	0 (-)	12 (100%) ****
AST and/or ALT > 40 U/Land/or gGT > 16 U/L	0 (-)	2 (15%) *
PLT < 150,000/mm^3^	0 (-)	0 (-)
ALP > 135 U/L and/or LDH > 240 U/L	0 (-)	9 (75%) ****
Headache and/or visual symptoms	0 (-)	7 (58.3%) ****
Presence of severe neurological symptoms	0 (-)	0 (-)
Pre-pregnancy body mass index (BM) (kg/m^2^)	22.5 ± 3.9	28.03 ± 8.5
Post-pregnancy body mass index (BM) (kg/m^2^)	27.2 ± 3.9	31.7 ± 7.9
Birth weight (g)	3410.08 ± 405.3	3168.3 ± 533.2
**Fetal sex**		
Female	6 (50%)	6 (50%)
Male	6 (50%)	6 (50%)

## Data Availability

The data presented in this study are available on request from the corresponding author.

## Supplementary Materials

The following supporting information can be downloaded at: https://www.mdpi.com/article/10.3390/ijms232112931/s1.
