# Supplementary material for: Preeclampsia Correlates with an Increase in Cannabinoid Receptor 1 Levels Leading to Macromolecular Alterations in Chorionic Villi of Term Placenta
Source: Int J Mol Sci. 2022 Oct 26;23(21):12931. doi: 10.3390/ijms232112931 (PMC9656520; doi:10.3390/ijms232112931)
Supplement: Supplementary file 1 [file ijms-23-12931-s001.zip › ijms-1979047-supplementary.pdf]

Preeclampsia Correlates with an Increase in Cannabinoid Receptor 1 Levels Leading to Macromolecular Alterations in Chorionic Villi of Term Placenta

Marta Lombó [1,2,†], Christian Giommi [1,†], Michela Paolucci [3], Valentina Notarstefano [1], Nina Montik [3], Giovanni Delli Carpini [3], Andrea Ciavattini [3], Antonio Ragusa [4], Francesca Maradonna [1,5,*], Elisabetta Giorgini [1] and Oliana Carnevali [1,5,*]

[1] Department of Life and Environmental Sciences, Università Politecnica delle Marche, 60131 Ancona, Italy
[2] Department of Molecular Biology, Faculty of Biology and Environmental Sciences, Universidad de León, 24071 León, Spain
[3] Department of Odontostomatological and Specialized Clinical Sciences, Università Politecnica delle Marche, 60020 Ancona, Italy
[4] Department of Obstetrics and Gynecology, Università Campus Bio Medico di Roma, 00128 Roma, Italy
[5] INBB—Consorzio Interuniversitario di Biosistemi e Biostrutture, 00136 Roma, Italy
* Correspondence: f.maradonna@staff.univpm.it (F.M.); o.carnevali@staff.univpm.it (O.C.)
† These authors contributed equally to this work.

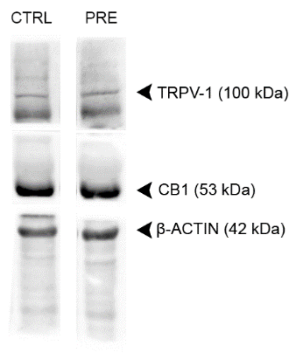

Figure S1. Representative experiment of western blot. Densitometric analysis of the band gray levels of CB1 (53 kDa) and TRPV-1 (100 kDa) in CTRL and PRE samples was performed using β-ACTIN (42 kDa) as internal control.
